# GAMMA-RAY: A Fully Automated and Rapid System for High-Dimensional Multi-Phenotype Analysis Considering Population Structure

**DOI:** 10.3390/biology15060496

**Published:** 2026-03-20

**Authors:** Taegun Kim, Jaeseung Song, Jong Wha Joanne Joo

**Affiliations:** 1 Department of Computer Science and Engineering, Dongguk University, Seoul 04620, Republic of Korea; taegun89@gmail.com; 2 Department of Life Sciences, Dongguk University, Seoul 04620, Republic of Korea; jaeseung6455@gmail.com; 3 Department of Computer Science and Artificial Intelligence, Dongguk University, Seoul 04620, Republic of Korea

**Keywords:** GWAS, multi-phenotype analysis, population structure correction, parallel computing, web-based interface

## Abstract

GWASs have successfully identified numerous genetic variants linked to complex traits, but traditional univariate approaches often fail to capture shared genetic architecture across multiple phenotypes. As the scale of genomic data continues to increase, the demand for more efficient multi-phenotype analysis methods has become particularly critical. In addition, the issue of population structure must also be properly addressed to ensure robust and unbiased results. Multivariate methods for multi-phenotype analysis, such as GAMMA, address this by combining linear mixed models with multivariate distance matrix regression to account for population structure; however, since these methods utilize computationally intensive models, developing efficient implementations is essential for practical analysis. Although GAMMA is a well-designed and effective tool, its original implementation relies on multiple programming environments and requires frequent data exchanges between components. These factors increase computational burden and complicate installation and execution for users unfamiliar with programming, making practical applications, particularly for high-dimensional datasets, challenging. Here, we present GAMMA-RAY, a high-performance C++ implementation that streamlines the computational pipeline, leverages parallel processing, and employs efficient matrix operations to achieve substantial reductions in runtime and memory usage. GAMMA-RAY provides both a user-friendly web-based interface for non-programmers and a standalone version for secure local execution. We further applied GAMMA-RAY to a yeast dataset and identified putative trans-eQTLs, in which several variants overlapped with previously reported cis- and trans-eQTLs. In addition, functional enrichment analysis revealed that the associated trans-eGenes are enriched, a conclusion consistently supported by biological annotation resources and underscoring the biological significance of these results.

## Simple Summary

Many biological traits are influenced by multiple genetic factors acting together, but traditional genetic studies often analyze one trait at a time. This approach can overlook important genetic effects that influence several traits simultaneously. To address this limitation, researchers have developed methods that analyze many traits together. However, existing approaches are often slow, require substantial computational resources, and are difficult to use in practice. In this study, we introduce GAMMA-RAY, a new software tool designed to make multi-trait genetic analysis faster, more efficient, and easier to use. GAMMA-RAY improves upon previous methods by reducing computation time and simplifying the analysis process while maintaining accurate results. Using genetic data from yeast, we demonstrate that GAMMA-RAY can effectively analyze complex patterns of gene activity. By providing both an easy-to-use web interface and a version that can be run locally, GAMMA-RAY makes advanced genetic analysis more accessible and supports future research aimed at understanding how genes jointly influence complex biological traits.

## 1. Introduction

Over the past decade, genome-wide association studies (GWASs) have been extensively conducted to discover genetic variants associated with various diseases and traits by examining the relationships between genetic variations across genomic and phenotypic outcomes [1,2,3,4,5,6]. Advances in next-generation sequencing technology have allowed for the collection of large-scale genomic data, including gene expression profiles, enabling the simultaneous analysis of thousands of phenotypes per individual. This development has facilitated more in-depth investigations into genomic regions that may affect multiple phenotypes at once. However, traditional GWAS approaches typically examine each trait separately, referred to as univariate tests, whereby the findings are reported independently, without accounting for potential interdependencies between traits [7,8]. This limitation underscores the need for innovative methods that can capture the complex relationships between multiple phenotypes and genomic variations.

Recently, multivariate approaches, which test the correlation between a variant and many phenotypes simultaneously, have been introduced. These techniques allow researchers to identify genetic variants that have pleiotropic effects, i.e., a variant affecting more than one trait or phenotype. Many genes are known to be regulated by only a few genomic regions, referred to as trans-regulatory hotspots (Wang et al., 2004; Cervino et al., 2005; Hillebrandt et al., 2005) [9,10,11], which strongly indicate the existence of master regulators of transcription. Multivariate analysis in expression quantitative trait loci (eQTL) studies can reveal such hotspots. Moreover, multivariate approaches are particularly valuable in uncovering shared genetic mechanisms and capturing the unmeasured aspects of complex biological networks, such as protein mediators, along with many phenotypes that might otherwise be missed when using a technique that focuses on a single phenotype or a few phenotypes (O’Reilly et al., 2012) [12]. Additionally, analyzing phenotypes together can improve the power to detect associations that might be missed when examining phenotypes individually. Single-cell or microbiome analysis often struggles with low abundance of specific cell types or microbial species and insufficient statistical power, issues that multivariate analysis can address.

Several multiple-test methods that jointly analyze multiple phenotypes are currently available. For instance, Nick et al. [13] used a mixed model to analyze gene co-expression (i.e., calculating gene co-expression while accounting for expression heterogeneity); however, due to its computational complexity, it is practical for only a small number of phenotypes. To analyze high-dimensional data, such as thousands of gene expressions for hotspot analysis, some researchers have utilized principal component analysis, cluster analysis, or factor analysis (Alter et al., 2000; Quackenbush 2001) [14,15]; however, these methods suffer from issues such as inadequate generalizability of principal components (Nievergelt et al., 2007; Aschard et al., 2014) [16,17]. As an alternative, Zapala and Schork (2012) [18] proposed multivariate distance matrix regression (MDMR) analysis, a technique based on distance matrices between phenotypes, which is relatively simple and directly applicable to high-dimensional multi-phenotype analysis. Unfortunately, this method does not consider the population structure and is known to produce many false positives [19]. Generalized analysis of molecular variance for mixed-model analysis (GAMMA) [20] is a more recently developed method, which incorporates a linear mixed model into MDMR and accounts for the population structure to enable accurate multi-phenotype analysis of high-dimensional data.

Although GAMMA has proven to be an effective tool for multivariate phenotype analysis, its usage in large-scale studies has been limited. It utilizes a linear mixed model, which is computationally burdensome, in addition to requiring numerous permutations to compute the *p*-value, as its summary statistics do not follow a standard statistical form. With the volume of genomic data continually growing, GAMMA remains impractical in its current form, which emphasizes the need for more efficient and scalable solutions. The original implementation of GAMMA employs both R (version 3.6.0) and Python (version 3.10), requiring multiple dependencies and frequent data exchanges between languages during runtime. This not only increases the computational burden but also complicates the installation and execution process, particularly for users unfamiliar with programming environments.

To overcome these challenges, we present GAMMA-RAY (Generalized Analysis of Molecular variance for Mixed models—Automated Rapid Analysis sYstem), a high-performance implementation of GAMMA developed entirely in C++ (version C++14). Unifying the computational pipeline into a single language, utilizing efficient functions and libraries of C++ that allow low-level control and optimized compilation, and eliminating intermediate input/output steps greatly reduces the execution time and system overhead. Furthermore, by leveraging parallel processing techniques, GAMMA-RAY can efficiently handle large-scale datasets with thousands of phenotypes. Moreover, we provide a web-based platform for GAMMA-RAY, available at https://cblab.dongguk.edu/GAMMA-RAY (accessed on 17 March 2026), which allows users to perform complex analyses directly through a web browser, without requiring advanced hardware or software setups. The system also includes interactive visualization features, facilitating intuitive exploration and interpretation of multivariate association results. Additionally, for users not willing to share their data with a third party, not only a downloadable source code but also a ready-to-use environment Docker image of GAMMA-RAY is available at https://github.com/DGU-CBLAB/GAMMA-RAY (accessed on 17 March 2026). Applied to a yeast [21] dataset comprising 1012 segregants genotyped at 42,052 SNPs with expression profiles for 5720 genes, we have demonstrated that GAMMA-RAY identifies 968 significant SNPs. Among these, 45 SNPs overlapped with previously reported cis-eQTLs, and 234 SNPs overlapped with known trans-eQTLs. Functional enrichment analysis further revealed that the associated trans-eGenes are enriched for fundamental cellular and regulatory processes consistent with known yeast genetic architecture, supporting a strong biological relevance of the GAMMA-RAY framework.

## 2. Materials and Methods

### 2.1. Fully Automated Web System for Multi-Phenotype Regression

**Figure 1:**
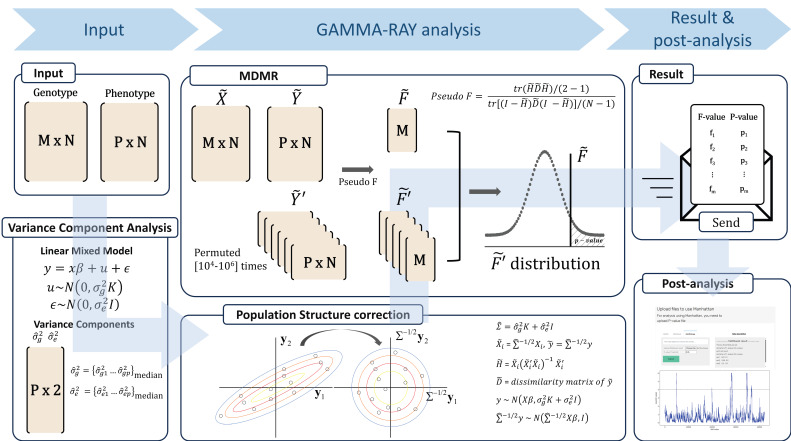
Overview of main processes in GAMMA-RAY. Genotype (M × N) and phenotype (P × N) matrices are uploaded by users. The pipeline performs variance component estimation and population structure correction, followed by MDMR for each variant. Statistical significance is assessed via permutation testing, and results are returned with visualization support.

GAMMA-RAY offers a web-based platform that facilitates complex multivariate analyses through a user-friendly interface. Users can initiate tasks directly via a browser, without installing any software or performing any manual configuration. The backend processes run seamlessly without user intervention, lowering the entry barrier for researchers who may not have programming experience. By simplifying access and improving usability, GAMMA-RAY extends the applicability of GAMMA to a wider scientific audience. Figure 1 presents the overall workflow of GAMMA-RAY. The genotype data are represented as an M × N matrix (with M genetic variants across N individuals), while the phenotype data are represented as a P × N matrix (with P phenotypes measured for the same N individuals). Upon input of these data by users, the platform first performs variance component analysis to estimate ${\hat{\sigma }}_{g}^{2}$ (the variance of the phenotype explained by genetic variation) and ${\hat{\sigma }}_{e}^{2}$ (the residual variance explained by environmental or other non-genetic factors). The median value of variance component estimates for P phenotypes is subsequently utilized for population structure correction through matrix transformation. Following this step, MDMR is performed for each genetic variant using multi-threading to compute the corresponding pseudo F-value. A permutation test is then conducted to generate the distribution of pseudo F-values, from which the associated *p*-value is obtained. The results are subsequently delivered to users via email. In addition, GAMMA-RAY provides a visualization tool to facilitate the interpretation of outcomes.

### 2.2. Client–Server Architecture

GAMMA-RAY is deployed using Apache Tomcat 9 as the application server. The web interface is constructed using HTML5, while CSS and JavaScript are utilized to enhance interactivity and usability. On the server side, the analysis engine is built using Java Servlet technology, which ensures a reliable and scalable client–server architecture.

Users can submit their input files and select analysis options directly through the web interface. The selected configurations are transmitted to the server, where data preprocessing and statistical analysis are performed automatically. Upon completion, a download link to the result files is automatically sent to the user via email.

**Figure 2:**
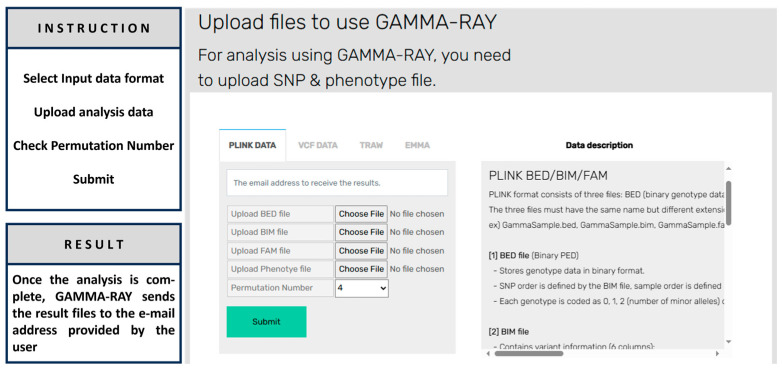
GAMMA-RAY system webpage interface.

Figure 2 displays the GAMMA-RAY web interface, where users can upload input data, configure analysis parameters, and view detailed descriptions of each step in the process.

### 2.3. Program Optimization in C++

The original GAMMA is developed using both R and Python, which necessitates frequent data exchanges between the two languages and often introduces an input/output overhead, thereby reducing performance. To overcome these limitations, we rebuild the program from the ground up in C++, creating GAMMA-RAY. This redesign not only removes the inefficiencies resulting from inter-language communication but also enables more streamlined and consistent code optimization. The integration of high-performance C++ libraries, such as Boost [22] and Eigen [23], significantly improves matrix computation efficiency and reduces runtime. Consequently, GAMMA-RAY offers a faster and more scalable solution for analyzing large genomic datasets while preserving the essential features of the original implementation.

### 2.4. Parallel Processing for SNP-Level Analysis

GAMMA-RAY is designed for high-throughput multi-phenotype association analysis and leverages parallel processing to improve computational efficiency. Because the same statistical computations are performed independently for each SNP, GAMMA-RAY splits the input genotype data into SNP-level units and processes them in parallel. The GAMMA algorithm involves multiple computational stages, each of which is complex and often comprises different procedures. To handle these stages efficiently, GAMMA-RAY leverages multi-threading techniques that utilize multi-core CPU architectures, enabling multiple SNP-level computations to run simultaneously within a single process. This design significantly reduces computation time compared with serial execution. Unlike other frameworks that rely on multiprocessing, GAMMA-RAY’s multi-threaded architecture minimizes memory overhead and facilitates faster inter-thread communication. By efficiently utilizing available CPU threads, GAMMA-RAY enhances the scalability and performance of genome-wide analyses.

### 2.5. Visualization Tools for Multivariate Results

GAMMA-RAY provides a visualization tool to assist in interpreting the outcomes of multi-phenotype association analysis. A set-based Manhattan plot displays the −log_10_(*p*-value) between a SNP and a set of phenotypes or gene expressions that the user wants to analyze across the genome. This allows users to visually identify significantly associated regions based on a predefined significance threshold and provides an intuitive overview of genome-wide signals of the multivariate test results. The visualization tool is designed to enhance the interpretability of high-throughput association results, and the generated figures can be downloaded for further analysis and reporting.

### 2.6. Local Deployment and Privacy Protection

Genomic data often contains sensitive personal health information, the uploading of which to external servers may pose privacy risks. To address these concerns, we have developed a downloadable version of GAMMA-RAY, which allows users to perform analyses entirely in their local environments. The complete source code, written in C++, is openly available on GitHub (commit ID: cb387c9, https://github.com/), along with a detailed manual that guides users through the compilation and setup process. This enables downloadable application users to run GAMMA-RAY on their personal computers or institutional servers without needing to share any data externally.

For users who prefer a ready-to-use environment, a Docker image of the GAMMA-RAY web system is also available. This image includes all necessary dependencies, eliminating the need for manual package installations or runtime environment configurations. With only a few commands, users can launch the GAMMA-RAY web interface locally or on the cloud. Step-by-step instructions for both compilation and Docker-based usage are included in the user manual. Furthermore, since the complete source code is provided, users are free to customize the software for their specific research needs. The Docker image, source code, and documentation are available at https://github.com/DGU-CBLAB/GAMMA-RAY (accessed on 17 March 2026).

## 3. Results

### 3.1. GAMMA-RAY Performance Improvement

**Figure 3:**
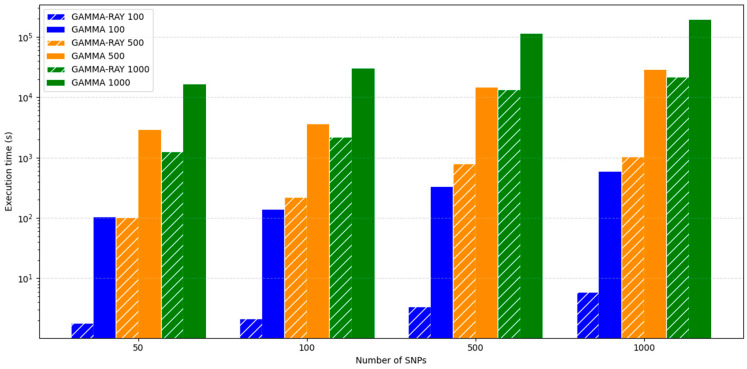
Log-scale execution time of GAMMA-RAY (hatched bars) and GAMMA (solid bars).

To evaluate the performance of GAMMA-RAY, we constructed datasets by selecting 50, 100, 500, and 1000 SNPs from the yeast dataset, and for each SNP set, we paired it with sample sizes of 100, 500, and 1000, resulting in a total of 12 experimental conditions. For each condition, results were obtained from a single execution, with up to 10,000 permutations performed for significance testing. Because identical input datasets and computational settings were applied, execution time and memory usage were expected to remain stable under these controlled conditions. Therefore, repeated runs were deemed unnecessary. Using these datasets, we conducted multivariate phenotype analysis on 5720 genes to assess computational efficiency. Under all tested conditions, GAMMA-RAY consistently outperformed GAMMA. The runtime reduced over 10-fold in most of the conditions with the performance gap becoming increasingly pronounced as the number of SNPs and samples grew. This substantial gain highlights GAMMA-RAY’s computational efficiency and scalability. Furthermore, across all experimental settings, GAMMA-RAY recorded shorter execution times than GAMMA, with the difference widening proportionally to the dataset size. Figure 3 shows log-scale execution times of GAMMA-RAY and GAMMA for different numbers of samples and SNPs.

To further validate the correctness of GAMMA-RAY, we conducted a quantitative comparison with the original GAMMA implementation across all experimental conditions. Spearman and Pearson correlations, as well as distributional analyses using the Kolmogorov–Smirnov test, confirmed a high concordance between the results of the two implementations (Supplementary Table S1), indicating that GAMMA-RAY faithfully reproduces GAMMA’s analytical outcomes.

**Figure 4:**
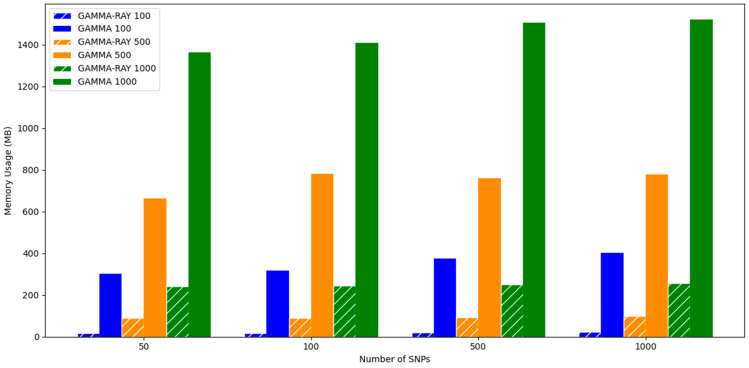
Memory usage (MB) of GAMMA-RAY (hatched bars) and GAMMA (solid bars).

In addition, we compared the memory usage of GAMMA and GAMMA-RAY. Figure 4 demonstrates that GAMMA-RAY is significantly more memory-efficient than GAMMA. These results underscore the advantages of GAMMA-RAY for analyzing large-scale datasets, highlighting its practical utility and effectiveness in high-throughput genomic studies.

All experiments were conducted on a server equipped with 267 GB of RAM and dual-socket Intel^®^ Xeon^®^ E5-2630 v4 CPUs (2.20 GHz), running CentOS Linux 7. To ensure a fair comparison, all computations were performed using a single CPU core.

### 3.2. GAMMA-RAY Identifies Genomic Regions Controlling Diverse Fundamental Biological Processes in a Yeast Dataset

**Figure 5:**
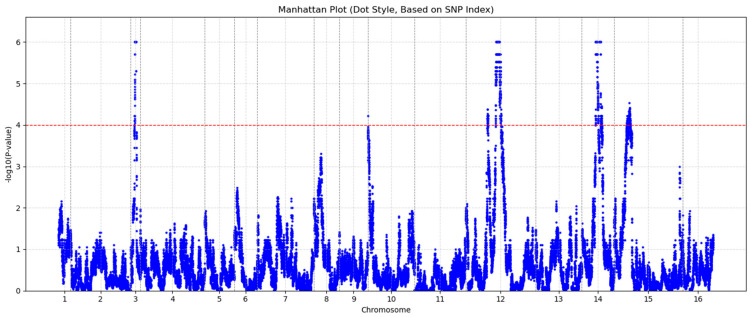
A set-based Manhattan plot of a yeast dataset. The multi-phenotype analysis results from GAMMA-RAY have been applied. The red dashed line indicates the *p*-value significance threshold.

After evaluating its performance on the previously mentioned subsets, we applied GAMMA-RAY to a yeast dataset, which comprises 1012 segregants genotyped at 42,052 SNPs and expression profiles for 5720 genes. A total of 968 significant SNPs were identified based on a significance threshold of *p* ≤ 1 × 10^−4^. Figure 5 shows a set-based Manhattan plot generated by GAMMA-RAY. Empirical *p*-values were calculated using permutation testing with up to 1,000,000 permutations to ensure stable estimation of small *p*-values. A significance threshold of *p* ≤ 1 × 10^−4^ was chosen as a conservative criterion, and all identified SNPs remained significant after applying Benjamini–Hochberg false discovery rate (FDR) correction, confirming the robustness of the findings.

A total of 968 significant SNPs were identified by GAMMA-RAY. Among these, 45 SNPs overlapped with previously reported cis-eQTLs, and 234 SNPs overlapped with known trans-eQTLs. The numbers of total eQTLs and their overlaps with known cis- and trans-eQTLs are summarized by chromosome in Table 1. Detailed information for all 968 significant SNPs is provided in Supplementary Table S2.

### 3.3. Identified Genomic Regions Are Controlling Diverse Fundamental Biological Processes

To investigate the biological functions of the genetic variants identified by GAMMA-RAY, we performed functional enrichment analysis using YeastEnrichr [24,25]. Genes located within a ±5 kb window of each (SNPs, eQTLs) of interest were used as input queries, and enrichment tests were conducted with Gene Ontology–Biological Process (GO-BP) and the Kyoto Encyclopedia of Genes and Genomes (KEGG) as reference pathways. Pathways with an adjusted *p*-value < 0.05 were considered significantly enriched [26,27,28,29,30].

**Figure 6:**
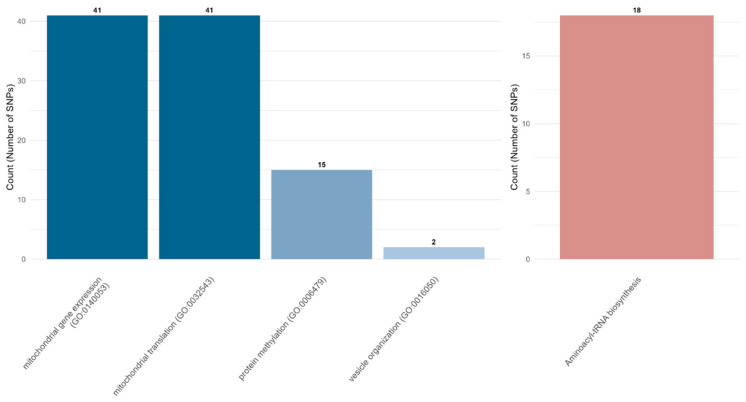
Representative biological processes controlled by each genomic region. The bar plots depicting the number of commonly enriched pathways for GO-BP (left panel) and KEGG (right panel) are shown. The full enrichment results are in Supplementary Tables S3 and S4.

Using this enrichment framework, we conducted functional enrichment analysis for their *trans*-regulating eGenes (Figure 6). After calculating the functional enrichments for each *trans*-eQTL region, we found a large overlapping enrichment of *trans*-eGene function in mitochondrial gene expression regulation and mitochondrial translation pathways across 41 *trans*-regulation variants. These results were driven by the existence of a single eGene, *YNL122C* (*mitochondrial 54S ribosomal protein bL35m*; *MRP35*) (Supplementary Table S3). We found that this gene is tightly regulated by the 41 SNPs on chr 14, showing the highly polygenic regulation effect within an identical locus of *MRP35*. Similar patterns were observed for the protein methylation pathway, which was driven by the *YNL092W*, the gene encoding an S-adenosylmethionine-dependent methyltransferase.

Other than the most highly overlapping pathways, we found that the 11 pathways were shown to be enriched by the pleiotropic regulation of two multi-functional genes (*YCL001W-A* and *YCL001W-B*) on chr 3. These two genes were involved in the diverse and fundamental biological pathways related to ribosomal assembly, biosynthesis, protein synthesis/disassembly, rRNA processing, gene expression regulation, and translation. Also, the KEGG pathway for aminoacyl-tRNA biosynthesis pathway was significantly enriched by both polygenic and pleiotropic regulations (both polygenic and pleiotropic effects of seven SNPs on chr3: *YNCC0008W* and *YNCC0009C*, polygenic effect of 11 SNPs on chr12: *YNCL0035C*).

## 4. Discussion

With the advancement of high-throughput technology, the production of genomic data is increasing at an unprecedented rate, leading to the accumulation of enormous amounts of data. Since the emergence of the missing-heritability problem, various approaches have emerged to explain and account for the missing genetic variance that is not captured by traditional GWAS. As part of these efforts, multi-phenotype analysis is conducted to leverage the shared genetic architecture across traits, improve power, and better understand the genetic basis of complex traits. GAMMA is one of the multi-phenotype analysis methods that corrects for spurious effects induced by the population structure, incorporating a linear mixed model. While GAMMA has been effective in detecting trans-regulatory hotspots from high-dimensional expression data, its practicality remains limited due to its excessive computational burden and the complexity of its execution process.

To address these issues, we developed GAMMA-RAY, which is coded entirely in C++ to streamline the execution pipeline and eliminate the inefficiencies introduced by the inter-language input/output overhead in the original version. By incorporating parallel processing and efficient matrix operations, GAMMA-RAY achieves significantly lower execution time and memory usage, enabling high-throughput analysis of large-scale genomic datasets. Performance tests conducted using yeast expression and genotype data under various sample and SNP sizes confirmed that GAMMA-RAY offers substantial improvements in speed and resource efficiency. In the most demanding scenario tested, GAMMA-RAY completed the analysis over 10 times faster than the original implementation while maintaining identical analytical results. In addition to performing better, GAMMA-RAY offers increased accessibility by providing both a web-based interface for users without programming experience and a standalone version for secure local use. This flexibility extends the tool’s applicability across different user groups and research settings.

By applying GAMMA-RAY to a yeast dataset, we identified 968 SNPs significantly associated with multivariate gene expression patterns, several of which align with previously reported cis- and trans-eQTLs. Functional enrichment analysis of the trans-eGenes showed strong concordance with the established genetic architecture of yeast, in which core biological processes are largely governed through trans-acting regulation [31,32]. Notably, the dense regulatory region surrounding MRP35 suggests that distal control of mitochondrial gene expression is mediated by a coordinated genetic module rather than a single causal variant. The presence of 41 SNPs on chromosome XIV associated with MRP35 expression further supports a polygenic regulatory architecture, whereby mitochondrial protein synthesis integrates multiple genetic signals instead of being controlled by a single master regulator.

A variety of multivariate GWAS tools exist and continue to be developed. MultiPhen [12] reverses the typical regression process by regressing genotype on phenotype to identify associations between a linear combination of phenotypes and an SNP. The advantage of this method is its simplicity and computational speed. Genome-wide Efficient Mixed Model Association (GEMMA) [33] and the matrix-variate linear mixed model (mvLMM) [34] use multiple trait variance component methods to model the covariance between phenotypes. These models consider the correlation between phenotypes while managing computational demands, making them suitable for a limited number of phenotypes in practice. Our method utilizes a distance matrix to conduct high-dimensional phenotype analysis and accounts for population structure. However, the method has several limitations. While GAMMA-RAY improves computational efficiency compared to the original GAMMA, substantial computational demands may still arise when analyzing very high-density datasets or performing large-scale permutation procedures. In addition, because GAMMA-RAY reports associations at the level of phenotype sets, further analyses are required to determine which specific phenotypes drive the observed SNP associations. Determining the most appropriate analysis method remains an open question, but researchers should consider dataset characteristics, phenotype dimensionality, and analysis goals when selecting among GAMMA-RAY and other multivariate GWAS tools. Furthermore, integrating results across multiple tools can facilitate a more comprehensive understanding of the complex genetic architecture underlying multiple phenotypes.

In this context, GAMMA-RAY offers a robust and scalable framework for high-dimensional genotype–phenotype association studies. Due to its user-friendliness and interpretability, we believe that it will be a valuable tool for large-scale analyses designed to clarify the genetic architecture of complex traits.

## 5. Conclusions

In this study, we developed GAMMA-RAY, a C++-based implementation of the GAMMA framework designed to address the computational limitations of multi-phenotype association analysis. By optimizing execution efficiency through parallelization and improved matrix operations, GAMMA-RAY substantially reduces runtime and memory usage while producing results identical to the original method.

Application to yeast genomic data demonstrated that GAMMA-RAY effectively identifies biologically meaningful trans-regulatory signals consistent with known genetic architectures. Together, these results indicate that GAMMA-RAY provides a scalable and accessible solution for high-dimensional genotype–phenotype association studies and will facilitate large-scale investigations into the genetic regulation of complex traits.

## Figures and Tables

**Table 1:** Significant eQTL regions identified using GAMMA-RAY.

CHR	Number of eQTLs	Known Cis-eQTLs	Known Trans-eQTLs
3	108	13	24
10	1	0	0
12	394	12	86
14	310	14	99
15	155	6	25

CHR: chromosome; number of eQTLs: number of eQTLs identified in the region; known eQTLs: number of previously reported eQTLs in the region.

## Data Availability

The processed yeast dataset analyzed in this study was obtained from Figshare: https://figshare.com/s/83bddc1ddf3f97108ad4 (accessed on 17 March 2026).

## Supplementary Materials

The following supporting information can be downloaded at: https://www.mdpi.com/article/10.3390/biology15060496/s1. Table S1: Pearson and Spearman correlations across sample sizes and SNP numbers. Table S2: Detailed information for all 968 significant SNPs identified in yeast. Table S3: GO Biological Process (GO-BP) enrichment results for trans-regulating eGenes across trans-eQTL regions. Table S4: KEGG pathway enrichment results for trans-regulating eGenes across trans-eQTL regions.

## Abbreviations

The following abbreviations are used in this manuscript:

GWAS	Genome-wide association studies
eQTLs	Expression quantitative trait loci
MDMR	Multivariate distance matrix regression
GAMMA	Generalized analysis of molecular variance for mixed-model analysis
GAMMA-RAY	Generalized Analysis of Molecular variance for Mixed models-Automated Rapid Analy-sis sYstem
GO-BP	Gene Ontology–Biological Process
KEGG	Kyoto Encyclopedia of Genes and Genomes
GEMMA	Genome-wide Efficient Mixed Model Association
mvLMM	matrix-variate linear mixed model
